# Dosimetry of [^177^Lu]Lu-DOTATATE in Patients with Advanced Midgut Neuroendocrine Tumors: Results from a Substudy of the Phase III NETTER-1 Trial

**DOI:** 10.2967/jnumed.124.268903

**Published:** 2025-03

**Authors:** Lisa Bodei, Marta Cremonesi, Mahila Ferrari, Erik S. Mittra, Harshad R. Kulkarni, Christophe M. Deroose, Rajaventhan Srirajaskanthan, John Ramage, Chiara Maria Grana, Francesca Botta, Matthias M. Weber, Matthias Miederer, Ryan Reddy, Daniela Chicco, Maurizio F. Mariani, Arnaud Demange, Jack L. Erion, Germo Gericke, Eric Krenning

**Affiliations:** 1 Department of Radiology, Memorial Sloan Kettering Cancer Center, New York, New York;; 2 Radiation Research Unit, Istituto Europeo di Oncologia, IRCCS, Milan, Italy;; 3 Medical Physics Unit, Istituto Europeo di Oncologia, IRCCS, Milan, Italy;; 4 Division of Molecular Imaging and Therapy, Oregon Health and Science University, Portland, Oregon;; 5 Theranostics Center for Molecular Radiotherapy and Molecular Imaging, Zentralklinik Bad Berka, Bad Berka, Germany;; 6 Nuclear Medicine, University Hospitals Leuven, Leuven, Belgium;; 7 Neuroendocrine Tumour Unit, ENETS Centre of Excellence, King’s College Hospital, London, United Kingdom;; 8 Department of Gastroenterology and Hepatology, Basingstoke and North Hampshire Hospital, Basingstoke, United Kingdom;; 9 Division of Nuclear Medicine, Istituto Europeo di Oncologia, IRCCS, Milan, Italy;; 10 Department of Endocrinology and Metabolism, I Medical Clinic, Johannes Gutenberg University of Mainz, Mainz, Germany;; 11 Clinic of Nuclear Medicine, Johannes Gutenberg University of Mainz, Mainz, Germany;; 12 Advanced Accelerator Applications, a Novartis company, Via Ribes, Italy;; 13 Advanced Accelerator Applications, a Novartis company, Geneva, Switzerland;; 14 Advanced Accelerator Applications, a Novartis company, New York, New York; and; 15 Cyclotron Rotterdam BV, Erasmus Medical Center, Rotterdam, The Netherlands

**Keywords:** [^177^Lu]Lu-DOTATATE, dosimetry, neuroendocrine tumors, peptide receptor radionuclide therapy, radiopharmaceutical therapy

## Abstract

This substudy of the phase III NETTER-1 trial evaluated [^177^Lu]Lu-DOTATATE (hereafter ^177^Lu-DOTATATE) for advanced midgut neuroendocrine tumors and aimed to assess dosimetry of a standard 4-cycle protocol and any potential relationship to toxicity. Change in tumor size by absorbed dose was an exploratory endpoint. **Methods:** Patients with locally advanced or metastatic, well-differentiated, midgut neuroendocrine tumors were enrolled in this substudy between August 2013 and January 2016. Patients were scheduled to receive 4 infusions of 7.4 GBq of ^177^Lu-DOTATATE for a cumulative injected activity of 29.6 GBq. After a ^177^Lu-DOTATATE infusion, whole-body planar images (4–6 time points for up to 7 d) and SPECT/CT images (at 24 and/or 48 h) were acquired, and absorbed and time-integrated activity coefficients were calculated to derive dosimetry. Blood and urine samples were used to determine the blood clearance and activity elimination rate. Tumor absorbed dose was derived using a sphere model, interpolating ^177^Lu dose factors on the basis of each lesion mass. Tumor size was assessed by measuring the longest and perpendicular dimensions on CT at measured time points. **Results:** Dosimetric assessments were evaluated in 20 patients. Organ dosimetry showed substantial interpatient variability. The predicted mean cumulative absorbed doses to kidneys and bone marrow were 19.4 (SD, 8.7) and 1.0 (SD, 0.8) Gy, respectively. Three patients had kidney doses between 28 and 33 Gy; 2 had grade 1 increased serum creatinine, and 1 showed no evidence of renal toxicity (up to 5 y of follow-up). Hematologic toxicity was primarily mild or moderate (grade 1–2) with no increase over time or association with cumulative absorbed dose. Tumor kinetics in 65 lesions demonstrated stable activity over time. Inter- and intrapatient variability was observed, and the median cumulative absorbed dose was 134 Gy (range, 7–2,218 Gy). Acknowledging the limitations of the imaging methods used and tumor volume assessments, we found no correlation between the best tumor size reduction and the absorbed dose, though most tumors (90%) shrank over the 72-wk study period. **Conclusion:** The dosimetry data support the findings that the standard treatment regimen with ^177^Lu-DOTATATE that includes personalized adjustments according to acute toxicity assessments is well tolerated and manageable.

## Visual Abstract

**Figure:**
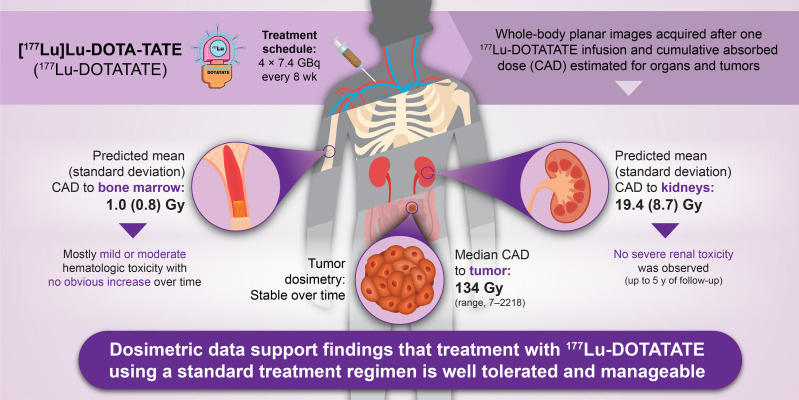

Peptide receptor radionuclide therapy (PRRT) using [^177^Lu]Lu-DOTATATE (hereafter ^177^Lu-DOTATATE) is indicated for the treatment of somatostatin receptor–positive neuroendocrine tumors (NETs) (Japan), gastroenteropancreatic NETs in patients 12 y and older (United States), or unresectable or metastatic, progressive, well-differentiated gastroenteropancreatic NETs in adults (Europe) (1–3). Efficacy and safety were established in 2 clinical studies: a phase I/II study conducted at the Erasmus Medical Center, Rotterdam (hereafter referred to as the Erasmus MC study), and the phase III NETTER-1 study (NCT01578239) (4–6). The results of the Erasmus MC study indicated that four 7.4-GBq infusions of ^177^Lu-DOTATATE could be safely administered without incidence of severe renal or bone marrow toxicity, provided that renal function and hematology assessments, as well as a treatment modification scheme reducing injected activity based on observed acute toxicity, were followed (4,7). This was confirmed in the NETTER-1 study (5,6).

Herein, we describe a NETTER-1 substudy that evaluated dosimetry (normal organs, whole body, and scintigraphically selected tumors) in patients with advanced midgut NETs treated with a standard treatment regimen of ^177^Lu-DOTATATE (up to four 7.4-GBq administrations adjusted according to observed acute toxicities). During this substudy, planar scintigraphy was used for dosimetry assessments. The potential limitations of this methodology compared with current imaging standards (8) are explored in the discussion.

## MATERIALS AND METHODS

The entire methods are available in the supplemental materials (available at http://jnm.snmjournals.org). The NETTER-1 substudy was a multicenter dosimetric study with a centralized data analysis derived from a homogeneous patient population (somatostatin receptor 2–positive advanced midgut NET, progressive under long-acting repeatable octreotide; additional details below).

### Patients and Treatments

Full patient eligibility criteria for the NETTER-1 study have been previously described (5). Briefly, eligible patients were adults with locally advanced or metastatic, well-differentiated (Ki-67 ≤ 20%), midgut NETs who had centrally confirmed disease progression (RECIST version 1.1) while taking long-acting repeatable octreotide (20–30 mg) every 3–4 wk. Patients had a positive uptake of ^111^In-DTPA-octreotide on planar scintigraphy (OctreoScan) in all target lesions and a Karnofsky Performance Score of at least 60.

Patients were treated using a standard protocol consisting of 4 infusions of 7.4 GBq of ^177^Lu-DOTATATE every 8 ± 1 wk (cumulative injected activity, 29.6 GBq), with injected activities adjusted or stopped on the basis of toxicity data. Patients also received 30 mg of long-acting repeatable octreotide every 8 wk, and for renal protection, a standard amino acid infusion was administered (5).

The primary objectives were to evaluate whole-body and organ radiation dosimetry of ^177^Lu-DOTATATE. Tumor dosimetry and response by absorbed dose were exploratory endpoints.

### Whole-Body Imaging and Dosimetry

Dosimetry assessments were performed using planar scintigraphy methodology routinely used at the time the study was conducted (9,10). After 1 infusion of ^177^Lu-DOTATATE, whole-body planar images were acquired for 4–6 time points for up to 7 d. SPECT/CT of the upper abdomen was performed at 24 and/or 48 h after ^177^Lu-DOTATATE administration to verify the activity distribution.

The geometric mean between the anterior and posterior images at each time point was calculated (11). Source organ radioactivity was determined at each time point, and cumulative activities were calculated (12). Absorbed dose values in target organs were calculated using time-integrated activity coefficients (13) of source organs using OLINDA/EXM version 1.0 (Hermes Medical Solutions) (14). A blood-based model was used to derive the cumulative activity in the red marrow and the corresponding absorbed dose (15,16).

For tumor dosimetry, baseline scintigraphy was used to select tumors that were easily delineated on imaging. Tumor doses were derived using the sphere model of OLINDA/EXM version 1.0 (14). The ^177^Lu dose factor values were interpolated for each lesion on the basis of its mass.

### Safety Assessments

Safety data were collected as previously described (5).

### Change in Tumor Size

The best tumor size change from baseline was assessed in individual lesions with dosimetric estimations. Tumor size was assessed by CT using 2 dimensions (longest [*L*] and perpendicular [*W*]) for each tumor per time point. Size change was based on the ratio of tumor area (*L* × *W*) at measured times (*T*_t_) to tumor area at baseline (*T*_bl_) and presented as a percentage where percentage tumor size change was ((*T*_t_/*T*_bl_) − 1) × 100%. Positive values indicate size growth, and negative values represent size reduction. Analyses were repeated using 1 dimension (short axis for lymph node lesions and long axis for other lesions). Tumor measurements were performed at 2–7 time points at 12-wk intervals until 72 wk after the first administration. The best tumor size change from baseline was defined as the greatest tumor size reduction or the lowest increase for those lesions that did not shrink at any time. However, the timings of dosimetry assessments were not the same for all lesions. Lesion uptake was assumed to be proportional to mass, resulting in a constant absorbed dose at all cycles for an equivalent injected activity. The cumulative absorbed dose was calculated for each target lesion. The correlation between tumor absorbed dose and effect was assessed by plotting the best percentage tumor size change from baseline versus the tumor cumulative absorbed dose.

### Trial Oversight

The study was approved by the institutional review board (or equivalent) and the ethics committee at each participating institution. It was conducted in accordance with Good Clinical Practice guidelines, as defined by the International Conference on Harmonisation, and all applicable local, national, and international regulations. All patients signed a written informed consent.

## RESULTS

### Patients and Treatments

This dosimetry substudy enrolled 30 patients between August 2013 and January 2016. Eight patients had been randomized to the ^177^Lu-DOTATATE arm in the NETTER-1 main study (5). Overall, 20 of 30 patients had evaluable dosimetric assessments, and baseline characteristics are shown in Table 1. The mean patient age was 57.4 y (SD, 11.2 y). Most patients (16/20 [80%]) had liver metastases. Fifteen patients received all 4 administrations of ^177^Lu-DOTATATE. Treatment was discontinued after 3 administrations in 3 patients (reasons were grade 2 lethargy, grade 3 white blood cell count decrease, and grade 3 generalized edema and fatal gastric obstruction). Two patients withdrew consent and discontinued the study, 1 after 2 administrations and 1 after the first administration. Median follow-up from the first ^177^Lu-DOTATATE administration was 62.9 mo (range, 9–89 mo).

**TABLE 1.** Baseline Characteristics of Evaluable Patients with Dosimetric Assessments

Characteristic	Evaluable (*n* = 20)
Sex	
Female	9 (45)
Male	11 (55)
Age (y)	57.4 (11.2)
Weight (kg)	79.2 (22.2)
Body surface area (m^2^)	1.90 (0.28)
Cumulative injected activity (GBq)	26.2 (6.6)
Metastases	
Liver	16 (80)
Bone	4 (20)
Number of ^177^Lu-DOTATATE administrations	
1	1 (5)
2	1 (5)
3	3 (15)
4	15 (75)
Timing of dosimetry	
Cycle 1	8 (40)
Cycle 2	6 (30)
Cycle 3	6 (30)
Median duration of follow-up (mo) (*n* = 19)*	62.9 (range, 9–89)
Number of target lesions (*n* = 17)^†^	65
Target lesion location	
Liver	33/65 (51)
Lymph node	16/65 (25)
Mesenteric	5/65 (8)
Paraaortic	5/65 (8)
Other^‡^	6/65 (9)
Peritoneum	3/65 (5)
Abdomen/bowel	3/65 (5)
Mesenteric mass	2/65 (3)
Bone	2/65 (3)
Mediastinal	2/65 (3)
Other^§^	4/65 (6)

* From the date of first ^177^Lu-DOTATATE administration (1 patient who discontinued immediately after first administration is excluded from the calculation).

† Three patients were nonevaluable.

‡ One each of paracardiac, retroperitoneal, inguinal, interportocaval, aortocaval, and paracaval.

§ One each in dorsomedial pleura, adrenal, paraspinal mass, and spleen.

Categoric variables are represented as number with percentage in parentheses, and continuous variables are represented as mean with SD in parentheses unless otherwise indicated.

### Organ Dosimetry

**FIGURE 1.**
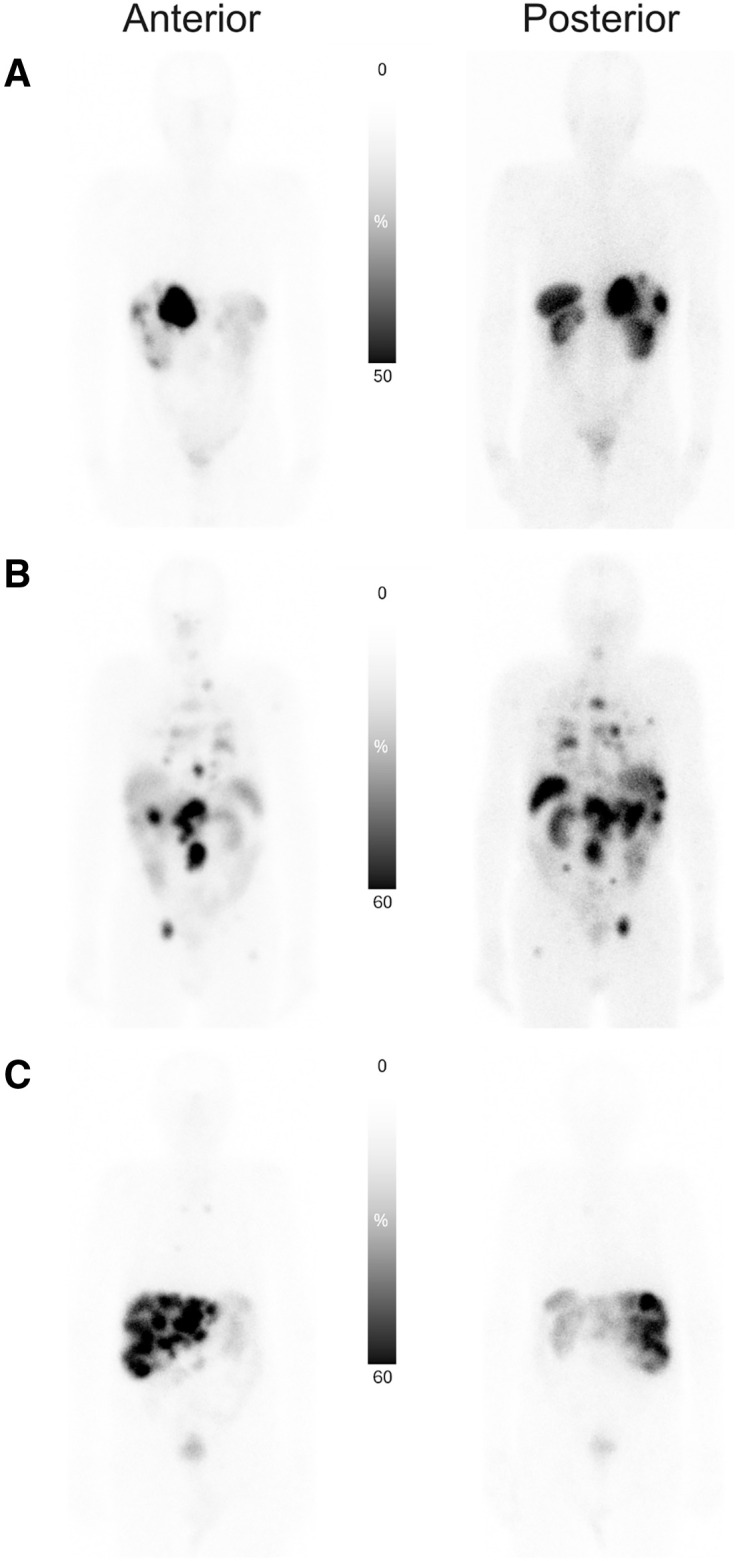
Planar images (anterior and posterior views at 24 h) showing typical biodistribution of ^177^Lu-DOTATATE. Linear intensity gray scale bars illustrate contrast enhancement, where bottom (absolute black) value represents specified percentage of original image’s maximum intensity. (A) Patient with low tumor burden. (B) Patient with multiorgan involvement. (C) Patient with predominately liver-only metastatic disease with high liver tumor burden.

**FIGURE 2.**
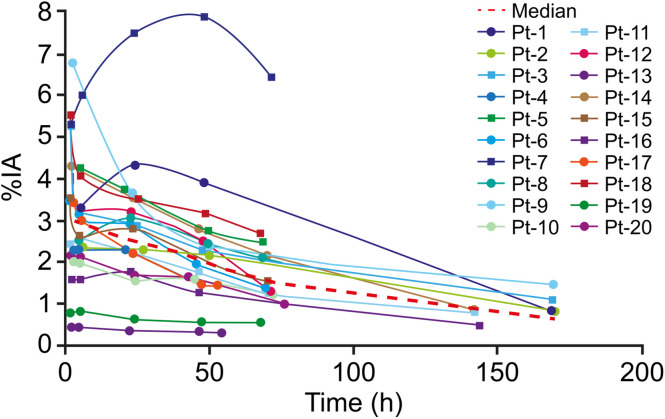
Time–activity curves in the kidneys after 1 administration of ^177^Lu-DOTATATE. Red dashed curve intersects median value of each time point. IA = injected activity; Pt = patient.

Planar images were collected at 4 (*n* = 3), 5 (*n* = 14), or 6 (*n* = 3) time points. The last time point was 6–7 d after treatment in 8 patients and 3 d after treatment for 12 patients. Whole-body image analyses showed that the spleen and kidneys had the highest ^177^Lu-DOTATATE activity, followed by the liver. Representative biodistribution patterns are seen in planar images, as shown in Figure 1. High interpatient variability was observed in organ dosimetry. In most patients, the time–activity curves for the assessed organs (spleen, kidneys, and liver) showed a biexponential trend. For some patients, the kidneys showed an initial phase of increasing uptake up to 24 h of ^177^Lu-DOTATATE administration, followed by a long-term elimination phase (Fig. 2). One patient showed a relatively high kidney injected activity profile, peaking around 8% at 48 h. This patient had stenosis of the ureter and withdrew from the study 1 mo after the first administration (no follow-up available). The overall effective half-life of the elimination phase ranged from 21 to 161 h. The mean time-integrated activity coefficient ranged from 0.3 h (red marrow) to 14.6 h (liver) and was 2.7 h (SD, 1.5 h) in the kidneys (Supplemental Table 1).

The estimated mean cumulative absorbed dose to normal organs was highest in the spleen and kidneys at 25.1 Gy (SD, 23.8 Gy) and 19.4 Gy (SD, 8.7 Gy), respectively. The absorbed dose to red marrow was estimated to be 1.0 Gy (SD, 0.8 Gy), and the overall total body absorbed dose was 1.6 Gy (SD, 0.8 Gy) (Table 2).

**TABLE 2.** Predicted Cumulative Absorbed Doses to Normal Organs and Target Lesions Calculated Per Unit of Activity and for Four Planned Administrations of 7.4 GBq (29.6 GBq total) of ^177^Lu-DOTATATE (*n* = 20)

	Absorbed dose per unit activity (Gy/GBq)		Absorbed dose for four 7.4 GBq (29.6 GBq in total) (Gy)	
Organ	Mean (SD)	Median (range)	Mean (SD)	Median (range)
Kidneys	0.65 (0.30)	0.68 (0.16–1.17)	19.4 (8.7)	19.3 (4.8–34.6)
Liver*	0.30 (0.23)	0.33 (0.05–2.06)	8.9 (6.7)	6.8 (1.6–23.7)
Red marrow from blood	0.04 (0.03)	0.03 (0.01–0.14)	1.0 (0.8)	0.7 (0.3–4.1)
Spleen	0.85 (0.80)	0.62 (0.18–3.61)	25.1 (23.8)	18.2 (5.4–106.9)
Urinary bladder wall	0.44 (0.18)	0.4 (0.2–0.8)	12.8 (5.3)	11.9 (4.5–22.6)
Total body	0.05 (0.03)	0.05 (0.01–0.1)	1.6 (0.8)	1.6 (0.4–3.1)
Tumor lesions^†^	7.6 (10.6)	4.5 (0.2–74.9)	224 (313)	134 (7–2,218)

* *n* = 18 (2 patients were nonevaluable because of high tumor burden).

† 65 lesions from 17 patients.

### Toxicities Relative to Absorbed Dose

Creatinine clearance values indicated that renal function remained stable and above the threshold for renal impairment (−20% per year starting from baseline) in most patients during treatment and follow-up (Supplemental Fig. 1). With up to 5 y of follow-up, no severe renal toxicity was observed. Three substudy patients had kidney doses between 28 and 33 Gy. Only 2 of these patients (absorbed doses of 28 and 32 Gy) had grade 1 increased serum creatinine, and both had grade 1 increased serum creatinine at baseline with other potential renal risk factors (controlled heart failure and type II diabetes mellitus, respectively). For both patients, serum creatinine remained grade 0–1 during treatment and follow-up, suggesting that it was unrelated to ^177^Lu-DOTATATE. The third patient with a kidney absorbed dose of 33 Gy showed no evidence of renal toxicity. The incidence of liver toxicities after treatment (grade 3 but no grade 4) was low (*n* = 4), and it was not persistent or considered clinically significant.

**FIGURE 3.**
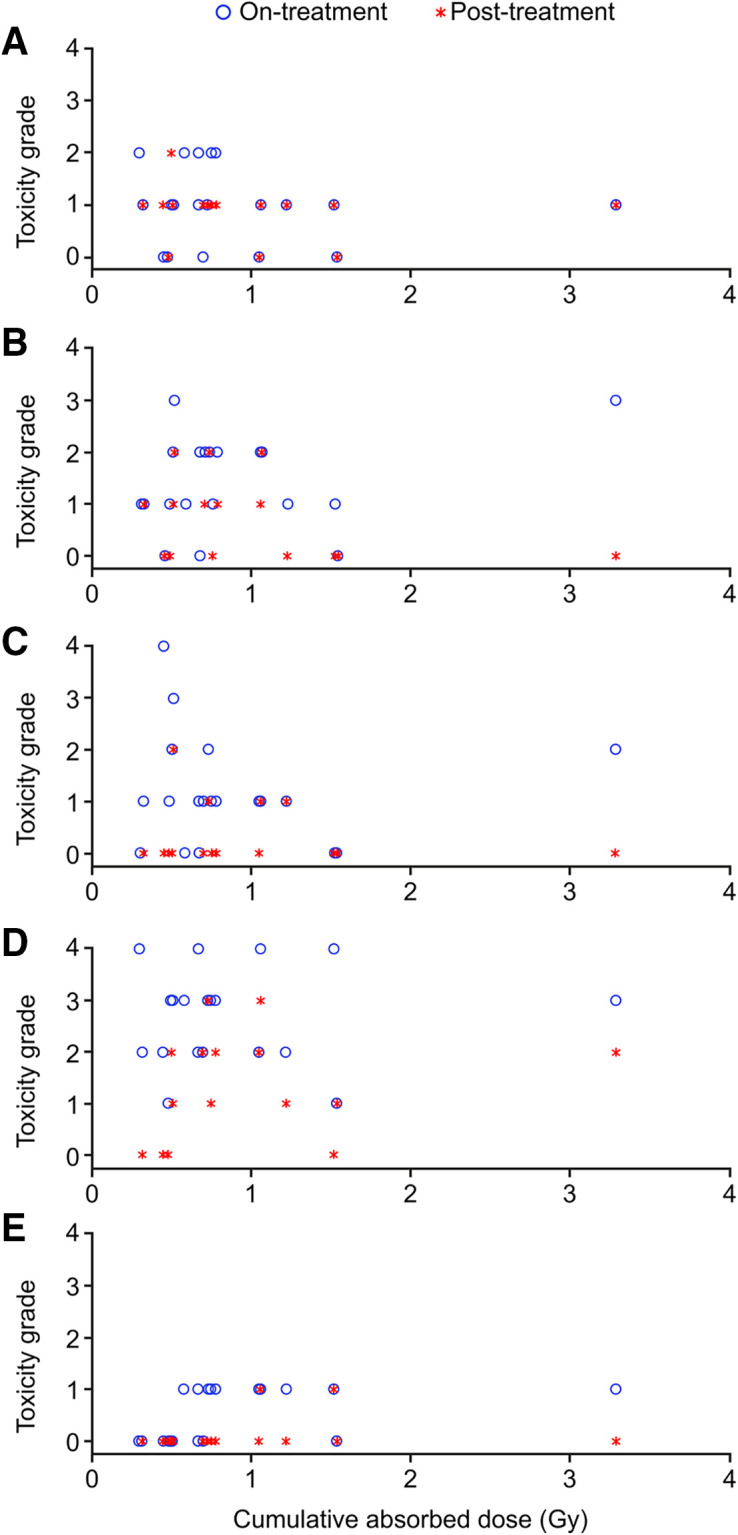
Bone marrow toxicity relative to cumulative absorbed dose. Point values are worst Common Terminology Criteria for Adverse Events version 4.03 score for bone marrow–related toxicity at any time during (○) or after (*) treatment at patient’s corresponding cumulative bone marrow absorbed dose. (A) Hemoglobin. (B) White blood cells. (C) Neutrophils. (D) Lymphocytes. (E) Platelets.

In general, acute hematologic toxicity was mild or moderate (grade 1 or 2), was not considered clinically relevant, and had no discernible pattern of increased toxicity over time (Supplemental Fig. 2). Overall, 2 of 20 (10%) patients experienced transient grade 3 leukopenia during treatment, which partially recovered during follow-up. There was no apparent association between the observed acute hematologic toxicity and spleen absorbed dose (data not shown). Receiving a higher absorbed dose did not result in higher-grade hematologic events. Hematologic events were grade 2 or less in patients with absorbed doses of more than 1 Gy, indicating no apparent association between bone marrow toxicity and cumulative absorbed dose (Fig. 3; Supplemental Table 2). One patient with disseminated bone lesions had a high cumulative red marrow dose of 3.2 Gy (Supplemental Fig. 3). This patient had grade 1 lymphopenia at baseline and grade 3 lymphopenia during treatment, which partially resolved to grade 2 during follow-up (Fig. 3B). Grade 4 lymphopenia during treatment (acute toxicity) was observed in 4 of 20 (20%) patients, who partially recovered during follow-up. Three of these patients had bone metastases, and 2 had lymphopenia (grade 2 and 3) at baseline. No patient with a dosimetry assessment developed myelodysplastic syndrome. One case from the nonrandomized substudy patients was reported, and along with the 2 cases reported in the NETTER-1 main study (6), the overall incidence of myelodysplastic syndrome was 3 of 133 (2.3%).

### Tumor Dosimetry

**FIGURE 4.**
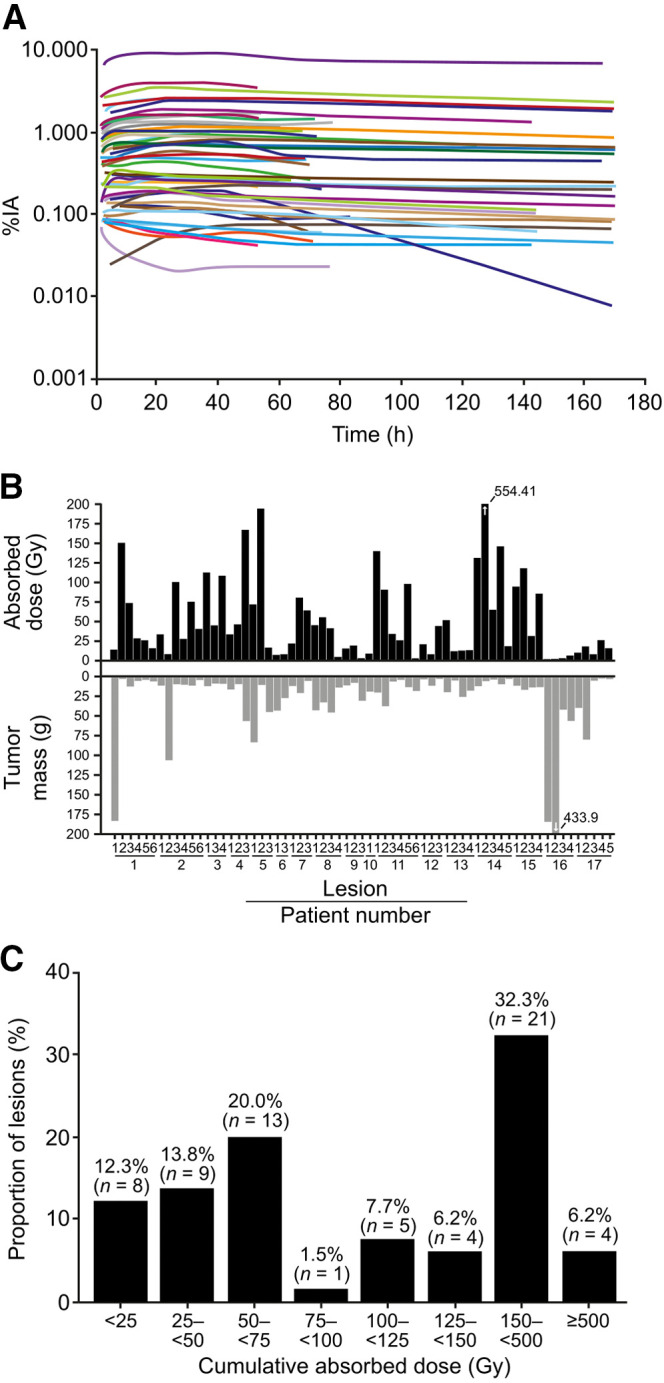
Tumor dosimetry. (A) Time–activity curves in target lesions after 1 administration of 7.4 GBq represented as percentage of injected activity (%IA) against time. (B) Absorbed dose after 1 administration of 7.4 GBq (top panel) and mass at time of dosimetry measurement (bottom panel) of all tumors evaluated (*n* = 65) in substudy patients with dosimetry (*n* = 17); arrows indicate values exceeding 200. (C) Frequency distribution of all tumors with dosimetry (*n* = 65) according to actual cumulative absorbed dose values.

Dosimetry in target lesions (*n* = 65, primarily liver lesions; Table 1) was evaluated in 17 patients. Nonevaluable patients had large lesions invading the liver that were indistinguishable from normal liver (*n* = 1), no visible uptake at the time of dosimetry analysis (*n* = 1, during the third ^177^Lu-DOTATATE administration), and a small lesion not within the resolution of the γ-camera (*n* = 1). Tumor dosimetry assessments were performed after the first (*n* = 7), second (*n* = 5), or third (*n* = 5) administration of ^177^Lu-DOTATATE. Tumor time–activity curves indicated a stable persistence of activity over time in all lesions except 1 (mesenteric lymph node), which showed a decline in activity after 40 h to less than 0.01% of injected activity at 160 h (Fig. 4A). Target lesion time–activity curves and absorbed doses showed high inter- and intrapatient variability (Figs. 4A and 4B). The estimated mean cumulative absorbed dose (assuming 4 full treatments) to all target lesions was 224 Gy (SD, 313 Gy), and the median was 134 Gy (range, 7–2,218 Gy) (Table 2). Comparing the absorbed dose from 1 treatment (7.4 GBq) with the tumor mass indicated a trend for larger tumors to have lower absorbed doses (Supplemental Fig. 4). The frequency distribution of cumulative absorbed doses in target lesions confirmed that ^177^Lu-DOTATATE had a variable uptake. However, high absorbed doses of at least 100 Gy or at least 50 Gy were observed in 52.3% (34/65) and 73.8% (48/65) of target lesions, respectively (Fig. 4C).

### Change in Tumor Size

**FIGURE 5.**
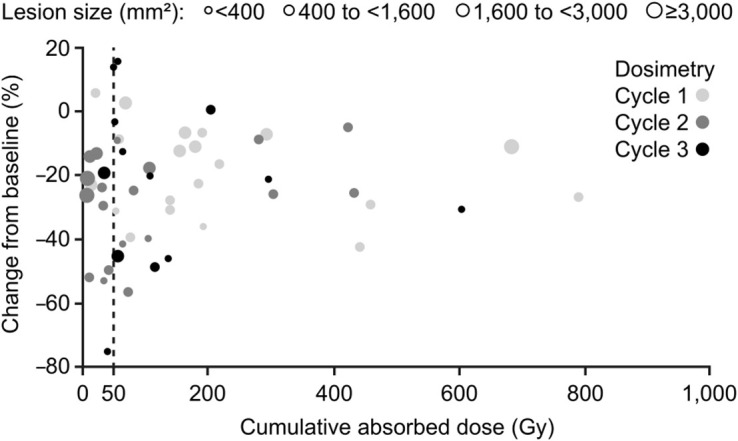
Best percentage tumor size change from baseline (assessed by area) at any time after treatment relative to cumulative absorbed dose of ^177^Lu-DOTATATE. Lesion size (mm^2^) corresponds to size at baseline (or first measurement).

Of the 17 patients with 65 lesions and dosimetric data, 15 (52 lesions) had dosimetric and CT measurements from at least 2 time points (including baseline values). Most lesions (38/52, 73%) received a cumulative absorbed dose of approximately 50 Gy or greater (range, 50–789 Gy); of those, tumor shrinkage was observed in 35 lesions (92%; range, −3.6% to −56.5%) (Fig. 5). No correlation was found between the best tumor size change from baseline (by area) and the cumulative absorbed dose, with changes observed across all dose values (Fig. 5). Regardless of the absorbed dose, 47 of 52 lesions (90%) had evidence of tumor shrinkage at some point in the 72-wk period. Similar results were obtained when the best tumor size change was assessed by diameter (Supplemental Fig. 5).

## DISCUSSION

In this NETTER-1 substudy, dosimetry was prospectively analyzed in patients with advanced midgut NETs, who received up to 4 administrations of 7.4 GBq of ^177^Lu-DOTATATE. It should be noted that patients were enrolled between August 2013 and January 2016, and imaging was performed using planar scintigraphy. Compared with today’s more advanced imaging methodologies, planar scintigraphy alone may not be the most accurate method for tumor dosimetry (17).

Concerning the historically identified critical organs (kidneys and bone marrow) (18,19), the results are consistent with the literature (7,19–23). Both the kidney and bone marrow mean dosimetry estimates (19.4 and 1.0 Gy, respectively) were below the commonly used conservative toxicity thresholds of 23 Gy for kidneys and 2 Gy for bone marrow (7,19,21). Three patients had kidney doses between 28 and 33 Gy, with only mild toxicity observed in 2 patients (both had renal risk factors at baseline). One patient exceeded 2 Gy in the bone marrow (3 Gy; bone lesions at baseline) and experienced grade 3 lymphopenia that had partially resolved at follow-up. Toxicity did not worsen over time. The favorable safety profile of ^177^Lu-DOTATATE was confirmed in the final NETTER-1 report, which included a safety follow-up of more than 5 y (6).

The acute toxicity of lymphocytes observed in 4 patients is not of major concern as ^177^Lu-DOTATATE mainly affects B lymphocytes, a subtype not critical for infection defense (24). Moreover, an increase in infectious diseases has not been established during PRRT with ^177^Lu-DOTATATE (24,25). The bone marrow dosimetry results should be considered alongside the inherent issues surrounding current blood-based and image-based methodologies for estimating bone marrow irradiation. Factors including the bone marrow matrix itself, the presence or absence of skeletal metastases, and patient characteristics including age, sex, and prior treatments have all been suggested as potential reasons for high interpatient bone marrow dosimetry variability and contrasting results regarding correlation with hematotoxicity (18,20,26–28).

Because of the small number of patients with renal toxicity, we cannot conclude whether modifying absorbed dose toxicity thresholds in response to the presence of other individually derived factors (renal risk factors [e.g., history of nephrotoxic chemotherapy or kidney impairment], hypertension, and intrinsic susceptibility to irradiation effects) that have been identified as possibly associated with PRRT toxicity (18) would be relevant. Overall, the absorbed dose to organs (primary endpoint) and organ toxicity profiles demonstrated that ^177^Lu-DOTATATE treatment is well tolerated, and these results are in line with the main study (5,29).

As per the literature, tumor absorbed doses and time–activity curves in this study were variable (30,31). The tumor absorbed dose tended to decrease as the tumor mass increased, which could be due to errors in estimating the mass of smaller tumors, tissue heterogeneity, lack of tumor vascularization, noncellular composition (fibrotic or necrotic tissue), pharmacokinetics, or other unknown factors. In our study, most lesions (73%) received a cumulative absorbed dose (approximately >50 Gy) sufficient to expect an observable effect on tumor size, and most (92%) did show some evidence of tumor shrinkage. Tumor size change was observed across all absorbed dose values, but no correlation existed between the best apparent tumor size change and the absorbed dose. Several reasons could explain this lack of correlation. First, the limits of planar dosimetry and the lack of specific correction for tumor dimension make it difficult to accurately determine the size of small tumors or the absorbed dose received (32–34). Clinical assumptions—that tumors are spheric, radioactivity uptake is homogeneous, and uptake is proportional to mass, resulting in a constant absorbed dose at all cycles for an equivalent injected activity—add to the complexity of the current analyses. Performing dosimetry measurements at any of the first 3 treatment cycles may have contributed to inaccuracy in estimated cumulative absorbed doses, and tumor makeup changes during treatment may affect the absorbed dose received by the tumor at subsequent cycles (30,31). Additionally, if the tumor dose at the first cycle is unknown, there may have been substantial tumor effects not captured when measuring at a later treatment cycle. True tumor volume regression may have been underestimated in many cases. Possible tumor-associated fibrosis or radiation-induced stromal fibrosis (31,35,36) can impact the accuracy of volume estimates using CT scans (37). Also, a detectable response to ^177^Lu-DOTATATE can be delayed (38,39). The main NETTER-1 study demonstrated that ^177^Lu-DOTATATE prolonged progression-free survival regardless of a detectable objective tumor response during treatment administration, indicating that a durable disease benefit goes beyond lesion shrinkage (40). Finally, our results are based on a relatively small patient number, and larger prospective studies may show different results.

There are very few prospective studies evaluating cumulative tumor absorbed dose and response to PRRT in the literature (30,32,41,42). A retrospective study by Jahn et al. reported a SPECT/CT-derived tumor absorbed dose versus tumor shrinkage (by volume) correlation in 23 patients with pancreatic NETs and 25 patients with small intestinal NETs (1 lesion of ≥2.2-cm diameter per patient) treated with ^177^Lu-DOTATATE, which was more pronounced for the pancreatic lesions, indicating that inherent tumor makeup and growth rate may affect the amount of tumor shrinkage observed for a given absorbed dose (30). In addition, a prospective study of personalized ^177^Lu-DOTATATE for progressive NETs reported no significant correlation between radiologic response and cumulative lesion absorbed dose 3 mo after treatment (22).

Ultimately, to propagate upfront dosimetry, there is a need for evidential studies to finally establish standardized protocols that rely on dosimetry as the basis for injected activity (26,43,44). Until pivotal trials are conducted that clearly show the benefit of personalized dosimetry for PRRT, a risk–benefit assessment that compares personalized treatment (adjusted based on dosimetric data) to a fixed injected activity treatment (adjusted taking into account acute toxicity) is not possible. To gain more insights, embedding dosimetric studies in early radiopharmaceutical therapy trials is an important strategy for novel radiopharmaceuticals.

## CONCLUSION

The findings of this prospective dosimetry study of the NETTER-1 trial support the tolerability and manageability of the standard PRRT protocol for ^177^Lu-DOTATATE administration of up to 4 cycles, which includes personalized injected activity adjustments based on toxicity assessments.

## DISCLOSURE

This study was funded by Advanced Accelerator Applications, a Novartis company. Lisa Bodei reports nonremunerated consultancies for Abdera, Amgen, Converge, Fusion, Great Point Partners, IBA, Ipsen, ITM, Novartis, Point Biopharma, RayzeBio, and SolveTx and an institutional research grant from Novartis. Erik Mittra has received honoraria as a consultant from Clarity, Curium, Lantheus, and TerSera and has research grants from Curium, ITM, Novartis, and RayzeBio. Christophe Deroose is a consultant for Advanced Accelerator Applications, a Novartis company, Immedica Pharma, Ipsen, IRE/IRE ELiT, Novartis, PSI CRO, Sirtex, and Terumo; his institution has received travel fees from GE HealthCare and Sirtex. Rajaventhan Srirajaskanthan reports grant funding from Ipsen and Novartis. Chiara Grana reports speaker honoraria and a travel grant from Advanced Accelerator Applications, a Novartis company; is a consultant for ITM; and reports a research grant from AIFA (Italian Health Ministry). Matthias Weber has received honoraria as a speaker or consultant from Ipsen and Novartis, as well as research funding from Ipsen and Merck. Matthias Miederer reports consultancies from Novartis, Roche, Telix, and Veraxa. Ryan Reddy reports nonremunerated consultancy for Advanced Accelerator Applications, a Novartis company. Daniela Chicco was an employee of Advanced Accelerator Applications, a Novartis company, and reports patents for diagnostic and therapeutic RLT agents. Maurizio Mariani was an employee of Advanced Accelerator Applications, a Novartis company. and reports patents for diagnostic and therapeutic RLT agents. Arnaud Demange is an employee of Advanced Accelerator Applications, a Novartis company. Jack Erion is an employee and shareholder of Advanced Accelerator Applications, a Novartis company, and has held patents, royalties, or other intellectual property. Germo Gericke was an employee of Advanced Accelerator Applications, a Novartis company. Eric Krenning (retired; Erasmus MC, Rotterdam, Netherlands) was a previous shareholder of Advanced Accelerator Applications, a Novartis company/BioSynthema. No other potential conflict of interest relevant to this article was reported.

## Notes

KEY POINTS **QUESTION:** What is the relationship between the absorbed dose (in healthy organs and tumors) and safety after standard treatment with ^177^Lu-DOTATATE, and is there a correlation between tumor absorbed dose and the best change in tumor size from baseline? **PERTINENT FINDINGS:** In a substudy of the phase III NETTER-1 trial, dosimetry analyses were conducted in 20 patients with advanced midgut NETs treated with 4 cycles of ^177^Lu-DOTATATE. Organ dosimetry showed high interpatient variability with no discernible relationship to the generally low to moderate renal and hematologic toxicities observed. Tumor dosimetry demonstrated a variable but stable persistence of activity over time and did not correlate with best change in tumor size from baseline. **IMPLICATIONS FOR PATIENT CARE:** The standard protocol for ^177^Lu-DOTATATE administration of up to 4 cycles is suitable for most patients, and further prospective analyses of dosimetry as a tool for personalizing PRRT are needed.
